# Uveitic crystalline maculopathy

**DOI:** 10.1186/s12348-015-0037-0

**Published:** 2015-02-14

**Authors:** Chris Or, Andrew W Kirker, Farzin Forooghian

**Affiliations:** Department of Ophthalmology & Visual Sciences, Faculty of Medicine, Eye Care Centre, Vancouver General Hospital, University of British Columbia, 2550 Willow Street, Vancouver, BC V5Z 3N9 Canada; Department of Ophthalmology, St. Paul’s Hospital, 1081 Burrard St., Vancouver, BC V6Z 1Y6 Canada

**Keywords:** Uveitic crystalline maculopathy, Crystalline maculopathy, Uveitis, Blood-retinal barrier

## Abstract

**Background:**

The purpose of this case report is to present a novel cause of crystalline maculopathy.

**Findings:**

A 52-year-old Japanese female presented with a 4-month history of decreased vision in the left eye. Best corrected visual acuity in the left eye was 20/40. Dilated fundus examination of the right eye was unremarkable, but that of the left eye demonstrated foveal yellow-green intraretinal crystals and mild vitritis. Optical coherence tomography of the left eye revealed small intraretinal fluid cysts and intraretinal crystals. Ultra-widefield fluorescein angiography was normal in the right eye, but that of the left eye demonstrated features of intermediate uveitis. There was no history or findings to suggest any cause for the crystals other than the uveitis.

**Conclusions:**

We propose that this may represent a novel category of crystalline retinopathy, termed uveitic crystalline maculopathy. We hypothesize that breakdown of the blood-retinal barrier as seen in uveitis may contribute to the deposition of crystals in the macula, although the precise composition of the crystals is unknown.

## Findings

### Introduction

Crystalline maculopathies are a group of disorders characterized by hyper-reflective crystalline deposits in the macula with a variety of recognized and proposed etiologies such as toxic, genetic, and degenerative causes [1]. Differentiation between these conditions may be difficult, and diagnosis usually requires a thorough history, including dietary intake and family history, and physical examination, with an emphasis on the nature and distribution of the hyper-reflective crystals. Fluorescein angiography (FA) and optical coherence tomography (OCT) have become useful tests in the evaluation of patients with crystalline maculopathy [1].

As our understanding of crystalline maculopathy has grown, several specific causes and risk factors have been identified, such as tamoxifen, canthaxanthine, oxalosis, Sjogren-Larsson syndrome, and West African crystalline maculopathy (WACM) [1]. Herein, we present a novel case of crystalline maculopathy associated with uveitis and an absence of recognized risk factors. We propose that this may represent a novel category of crystalline maculopathy, termed uveitic crystalline maculopathy.

### Case report

**Figure 1:**
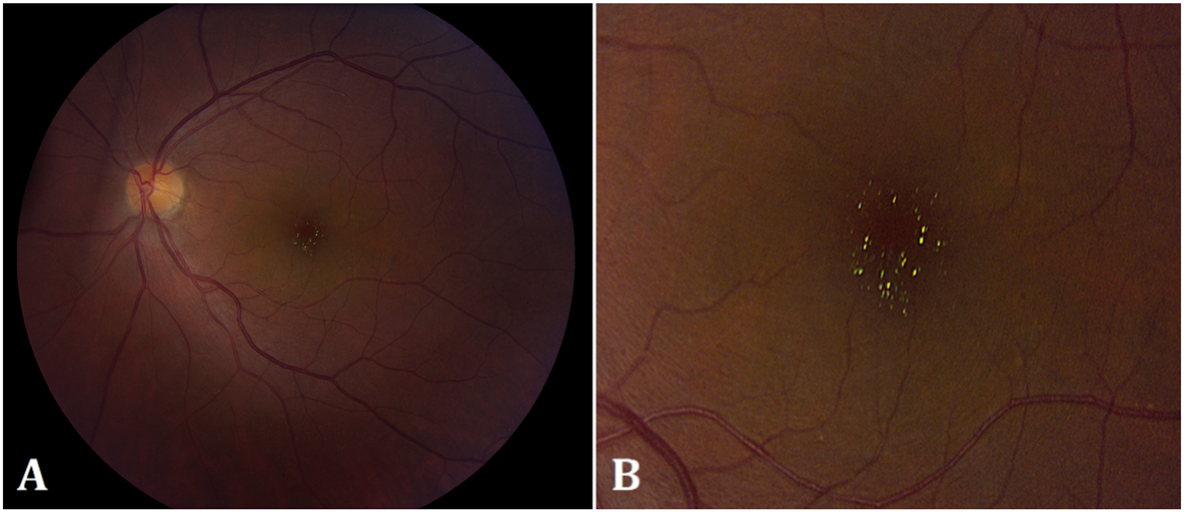
**Color photographs of the left eye of the patient with uveitic crystalline maculopathy.** Numerous foveal yellow-green hyper-reflective crystals could be observed in **(A)** and in the magnified view in **(B)**.

**Figure 2:**
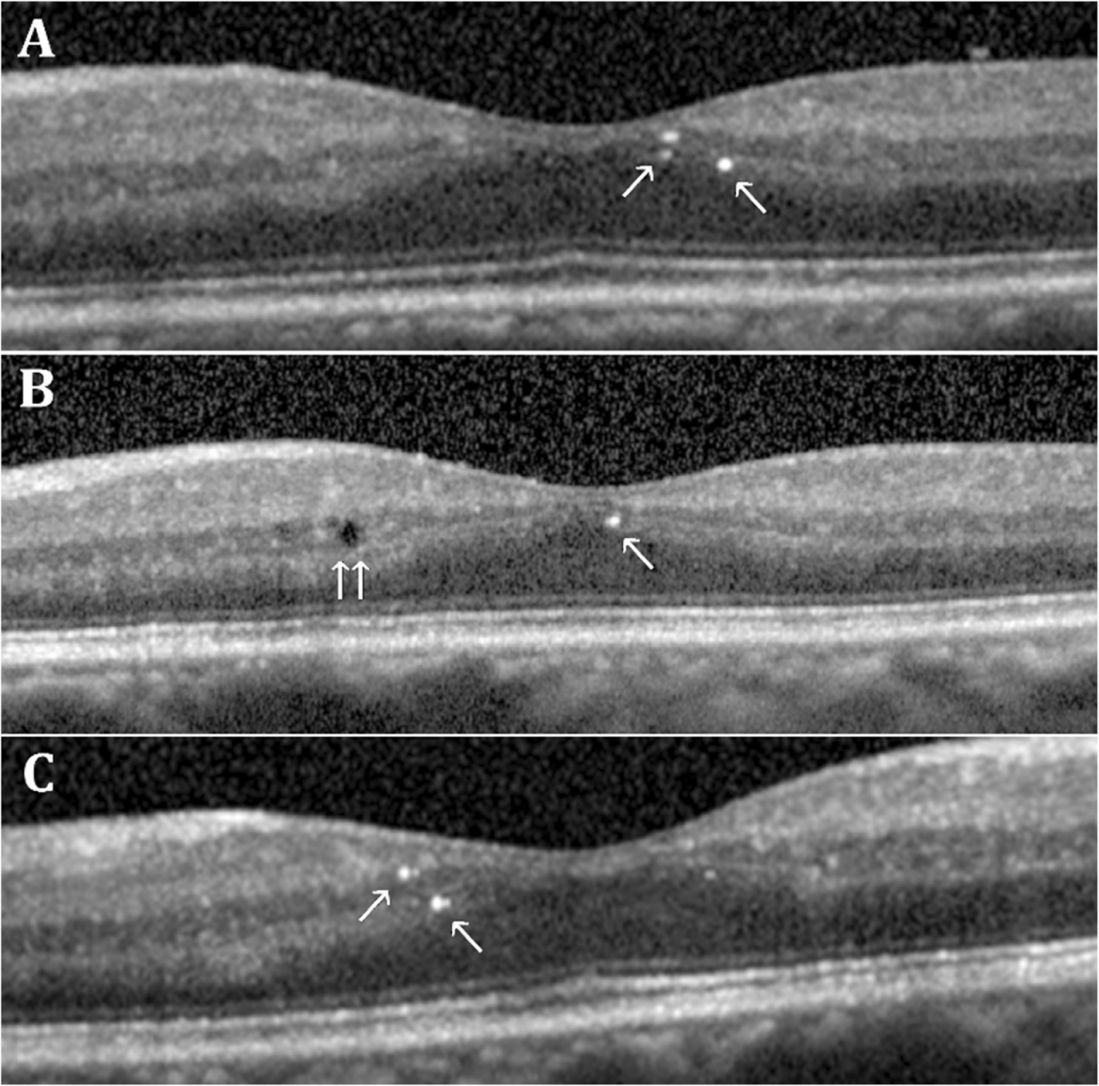
**Spectral-domain optical coherence tomographic scans of the patient with uveitic crystalline maculopathy.** Hyper-reflective intraretinal crystals (single arrows) of varying sizes could be seen dispersed in the layer of Henle and the inner nuclear layer **(A, B, C)**. Intraretinal cysts (double arrows) could also be observed in the inner retinal layers **(B)**.

**Figure 3:**
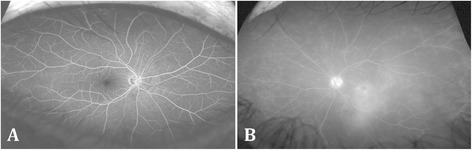
**Ultra-widefield fluorescein angiogram (UWFA) of the patient with uveitic crystalline maculopathy.** UWFA of the right eye is normal **(A)**. UWFA of the left eye revealed disc leakage, petaloid macular edema, and diffuse peripheral retinal leakage, consistent with intermediate uveitis **(B)**.

A 52-year-old Canadian-born Japanese female presented with a 4-month history of decreased vision of the left eye (OS). Past history revealed calcific rotator tendinopathy, which required two barbotage treatments. Best corrected visual acuity (BCVA) was 20/20 in the right (OD) and 20/40 OS. Intraocular pressure was 20 mmHg OD and 22 mmHg OS. Slit-lamp examination revealed posterior chamber intraocular lens implants bilaterally. Dilated fundal examination of the right eye was unremarkable, but that of the left eye demonstrated foveal yellow-green intraretinal crystals (Figure 1) and mild vitritis. OCT of the left eye revealed small intraretinal fluid cysts and intraretinal hyper-reflective lesions in Henle’s layer and inner nuclear layer, corresponding to the clinically observed crystals (Figure 2A,B,C). OCT of the right eye was normal. Ultra-widefield fluorescein angiography was normal in the right eye (Figure 3A), but that of the left eye showed disc leakage, petaloid macular edema, and diffuse peripheral retinal leakage (Figure 3B), consistent with intermediate uveitis. No peripheral retinal detachment, vascular telangiectasia, or retinal pigment epithelial abnormalities were observed in either eye.

There was no family history of African ancestry or a family history of crystalline maculopathy. There was no history of tamoxifen, canthaxanthine, methoxyflurane, and nitrofurantoin exposure; IV drug use; or kola nut consumption. She admitted to eating a diet high in oxalate (spinach, kale, chard). CT of the chest, purified protein derivative (PPD) test for tuberculosis, angiotensin-converting enzyme levels for sarcoidosis, FTA-ABS for syphilis, CBC, urinalysis, serum creatinine, ionized calcium, and 24-h urine oxalate were all normal.

The patient was treated with posterior subtenon triamcinolone (40 mg, Triesence, Alcon Laboratories, Inc., Fort Worth, TX, USA), with little improvement. The patient was then treated with intravitreal triamcinolone (4 mg, Triesence), and the intraretinal fluid subsequently resolved. BCVA improved to 20/30 OS, but the crystals remained.

### Discussion

Our case represents the first described case of uveitic crystalline maculopathy. The clinical characteristics of this case, particularly the characteristics and distribution of the crystals observed, are similar to those of West African crystalline maculopathy. WACM was first reported in 2003 by Sarraf et al. in elderly members of the Igbo Tribe in Nigeria and was characterized by bilateral asymptomatic yellow-green hyper-reflective crystals in the superficial fovea, localized to the layer of Henle on OCT, and hypothesized to be related to kola nut ingestion [2]. Similar reports have subsequently been described in several other regions in West and East Africa, and the role of kola nut ingestion in the pathogenesis of this condition remains under question [3-5]. Browning suggested that diabetes mellitus may contribute to the pathogenesis of WACM, as every patient in his case series had diabetes and the distribution of crystals appeared to correlate with areas of diabetic exudate and retinal edema [5]. This was supported by a subsequent case series by Rajak et al. who noted that all patients in their case study had diabetes or another coexistent retinal pathology that could result in retinal ischemia, such as sickle cell retinopathy or branch macular vein occlusion [3]. It was then postulated that disruptions to the blood-retinal barrier secondary to retinal ischemia in susceptible patients could result in macular crystal formation in WACM, likely as a result of exudation of pre-formed crystals or precipitation from exudated serum [3]. We hypothesize that a similar mechanism underlies the pathogenesis of uveitic crystalline maculopathy, as breakdown of the blood-retinal barrier is fundamental to the development of uveitis. The fact that crystals could only be seen in the eye with uveitis in our case emphasizes its involvement in the pathogenesis of uveitic crystalline maculopathy.

We propose that this case represents a novel category of crystalline maculopathy, one in which uveitis is directly involved in the pathogenesis of the condition. However, the composition of the crystals is still unknown. Further studies are required to fully elucidate the pathogenesis and etiological basis of uveitic crystalline maculopathy.
